# Correlation between long non-coding RNA MAFG-AS1 and cancer prognosis: a meta-analysis

**DOI:** 10.3389/fonc.2023.1286610

**Published:** 2023-12-07

**Authors:** Guangyao Lin, Huicong Liu, Jingyu Lin, Xiyu Liu, Lianwei Xu

**Affiliations:** Department of Gynecology, Longhua Hospital, Shanghai University of Traditional Chinese Medicine, Shanghai, China

**Keywords:** lncRNA MAFG-AS1, malignancies, meta-analysis, prognosis, review

## Abstract

**Background:**

MAF transcription factor G antisense RNA 1 (MAFG-AS1), a novel long non-coding RNA discovered recently, was proved to be useful in predicting malignancy prognosis. Nevertheless, its association with cancer prognosis has been inconsistent. Therefore, this meta-analysis aimed to explore the clinicopathological and prognostic significance of MAFG-AS1 in diverse carcinomas.

**Methods:**

Studies focused on MAFG-AS1 expression as a prognostic role in cancers were thoroughly searched in six electronic databases. The value of MAFG-AS1 in malignancies was assessed by hazard ratios (HRs) or odds ratios (ORs). Additionally, the GEPIA database was utilized to further strengthen our conclusion.

**Results:**

A total of 15 studies involving 1187 cases and nine types of cancers were recruited into this meta-analysis. High MAFG-AS1 expression was significantly related to advanced tumor stage (OR = 0.52, 95%CI [0.39, 0.69], P < 0.00001), earlier lymph node metastasis (OR = 3.62, 95%CI [2.19, 5.99], P < 0.00001), worse tumor differentiation (OR = 0.64, 95%CI [0.43, 0.95], P = 0.03), and poor overall survival (HR = 1.94, 95%CI [1.72, 2.19], P < 0.00001). No significant heterogeneity and publication bias was detected across studies. Meanwhile, MAFG-AS1 was significantly elevated in ten kinds of cancers based on the validation of the GEPIA database.

**Conclusion:**

The results of this meta-analysis indicated that high MAFG-AS1 expression is dramatically correlated with unfavorable prognosis in cancers. MAFG-AS1 may be served as a promising biomarker for malignancies.

## Introduction

Cancer is a major threat to human health worldwide. According to the cancer statistics published by the American Cancer Society recently, there were approximately 0.6 million cancer deaths and 1.95 million newly diagnosed cancer cases in the United States in 2023 (1). Furthermore, in China, an estimated 3 million cancer-related deaths and 4.5 million new cancer cases occurred during 2020, accounting for 30.2% of all cancer deaths in the world, which were significantly higher than those in the United States and thus remarkably increased the cancer and economic burden in China (2). Although breakthroughs in diagnostic techniques such as circulating tumor DNA, circulating tumor cells, and positron emission tomography/computed tomography have notably improved cancer surveillance, the mortality of many malignancies has not been considerably reduced (3–5). The GLOBOCAN 2020 estimated that there will be 28.4 million cancer cases in 2040 (6). Therefore, exploring new cancer biomarkers to detect early-stage cancer and determine the prognosis of cancer patients is rather imperative and rewarding (7).

Long non-coding RNAs (lncRNAs) which exceed 200 nucleotides in length and lack protein-coding potential have been identified in an extensive range of biological processes, like regulating gene expression and shaping nuclear structure (8). More recently, mounting evidence suggested that lcnRNAs were highly associated with prognosis in various malignancies and may be novel targets for cancer detection and therapy (9, 10). For example, the MAF transcription factor G antisense RNA 1 (MAFG-AS1), a novel lncRNA with a transcript size of 1914bp, is located on chromosome 17q25.3 (11, 12). Clinical studies had demonstrated that elevated expression of MAFG-AS1 could accelerate the progression of diverse kinds of cancers, including breast cancer, bladder cancer, hepatocellular carcinoma, lung cancer, gastric cancer, colorectal cancer, glioblastoma, ovarian cancer, prostate cancer, pancreatic cancer, and esophageal squamous cell cancer (ESCC) (12). Basically, overexpression of MAFG-AS1 is closely connected to higher histological grade, lymph node metastasis (LNM), larger tumor size, and shorter overall survival (OS) in many human cancers (13). Simultaneously, accumulating experiments indicated that MAFG-AS1 participated in various biological effects, such as promoting migration, proliferation, and epithelial-mesenchymal transition (EMT), along with inhibiting apoptosis of carcinoma (12). Collectively, MAFG-AS1 appeals to a wide range of clinicians and may serve as a potential predictor of carcinoma prognosis.

Although a considerable number of clinical studies have investigated the correlation between MAFG-AS1 and cancer prognosis, several variables concerning MAFG-AS1 in malignancies have generated controversial results. For example, Li et al. proved that high MAFG-AS1 expression in bladder cancer was obviously correlated with larger tumor size (≥ 3 cm), but not with tumor stage (14). Instead, Sun et al. also estimated MAFG-AS1 expression in bladder cancer and demonstrated that elevated MAFG-AS1 expression was closely associated with tumor stage (P < 0.05), but not with larger tumor size (≥ 3 cm) (15). Furthermore, regarding breast cancer, Di et al., based on 54 cases, confirmed that overexpression of MAFG-AS1 was significantly related to LNM (P < 0.05) (11), which was contrary to Feng’s study (16). Consequently, the significance of these associations may be insufficiently evaluated, due to the small sample sizes of individual study. Therefore, this meta-analysis was conducted to quantitatively estimate the prognostic significance of MAFG-AS1 in various cancers.

## Materials and methods

This study was performed following the preferred reporting program of the systematic review and meta-analysis (PRISMA) (17).

### Search strategy

Six databases, including Web of Science, Springer, Cochrane Library, PubMed, Scopus, and EBSCO were thoroughly searched from inception up to August 22, 2023, for eligible studies. The search keywords were as follows: MAF transcription factor G antisense RNA 1, long non-coding RNA MAFG-AS1, lncRNA MAFG-AS1, and MAFG-AS1. Besides, the references of retrieved records were evaluated carefully to screen more potentially relevant records.

### Inclusion and exclusion criteria

The inclusion criteria for this study were as follows: (1) randomized controlled trials or retrospective studies; (2) patients were divided into high MAFG-AS1 expression group and low MAFG-AS1 expression group; (3) studies provided OS, disease-free survival (DFS), or clinicopathologic parameters (e.g., lymph node metastasis, tumor differentiation, tumor size, tumor stage, gender, and age of patients) at least; (3) malignancies were solid tumor; (4) patients had not been treated with chemotherapy or radiotherapy prior to surgery; (5) no ethnical and geographical restrictions.

The exclusion criteria were as follows: (1) studies failed to report sufficient data (e.g., without clinicopathologic parameters, OS, or DFS); (2) data from public databases, duplicate publications, cellular-based experiments, reviews, animal experiments, non-solid tumor, retracted articles; (3) studies were not published in English.

### Data extraction and quality assessment

The first two authors independently summarized the major characteristics of eligible studies after being screened by our inclusion and exclusion criteria. The following items were recorded: the author’s last name, publication year, types of cancer, sample size capacity, number of patients from two groups, detection technique, prognostic variables (e.g., OS and DFS), corresponding hazard ratio (HR) and 95% confidence interval (CI), clinicopathologic parameters, and data extraction method for OS, along with follow-up time. If the study only showed Kaplan-Meier curves without listing accurate HR and 95% CI, then the HR and 95% CI were extrapolated based on Engauge Digitizer 4.1 software indirectly (18). Any disagreement was resolved after prompt discussion with the corresponding author.

### Validation of MAFG-AS1 expression in diverse cancers

We determined the expression of MAFG-AS1 in normal tissues with diverse tumor tissues utilizing Gene Expression Profiling Interactive Analysis (GEPIA) which comprised 9736 tumors clinical samples based on GTEx and TCGA data, and it has been widely adopted to validate meta-analysis results (19–21). P < 0.01 was regarded as significantly statistical.

### Statistical analysis

Review Manager 5.3 software were adopted for statistical analysis. EndNote 20.2 software was utilized for document management. We calculated the association between MAFG-AS1 expression and clinicopathologic parameters across studies using pooled odds ratios (ORs) and 95% CIs. Besides, HR and 95% CIs were estimated to investigate the correlation between MAFG-AS1 expression and the OS of various malignancies. Moreover, the fixed- or random-effects models were applied to determine the summarized OR or HR and 95% corresponding CIs based on between-study heterogeneity. If I^2^ ≥ 50%, the random-effects model was selected. Otherwise, the fixed-effects model was utilized. P < 0.05 was deemed as statistically significant. Furthermore, sensitivity analysis was conducted by sequentially omitting individual studies to appraise whether the results were evidently impacted by individual study if at least five studies were involved. Subsequently, Begg’s and Egger’s tests were performed using Stata 15.1 software to objectively demonstrate publication bias, if at least ten studies were included, and P > 0.05 was supposed that there was no publication bias existing among studies.

## Results

### Included articles

**Figure 1:**
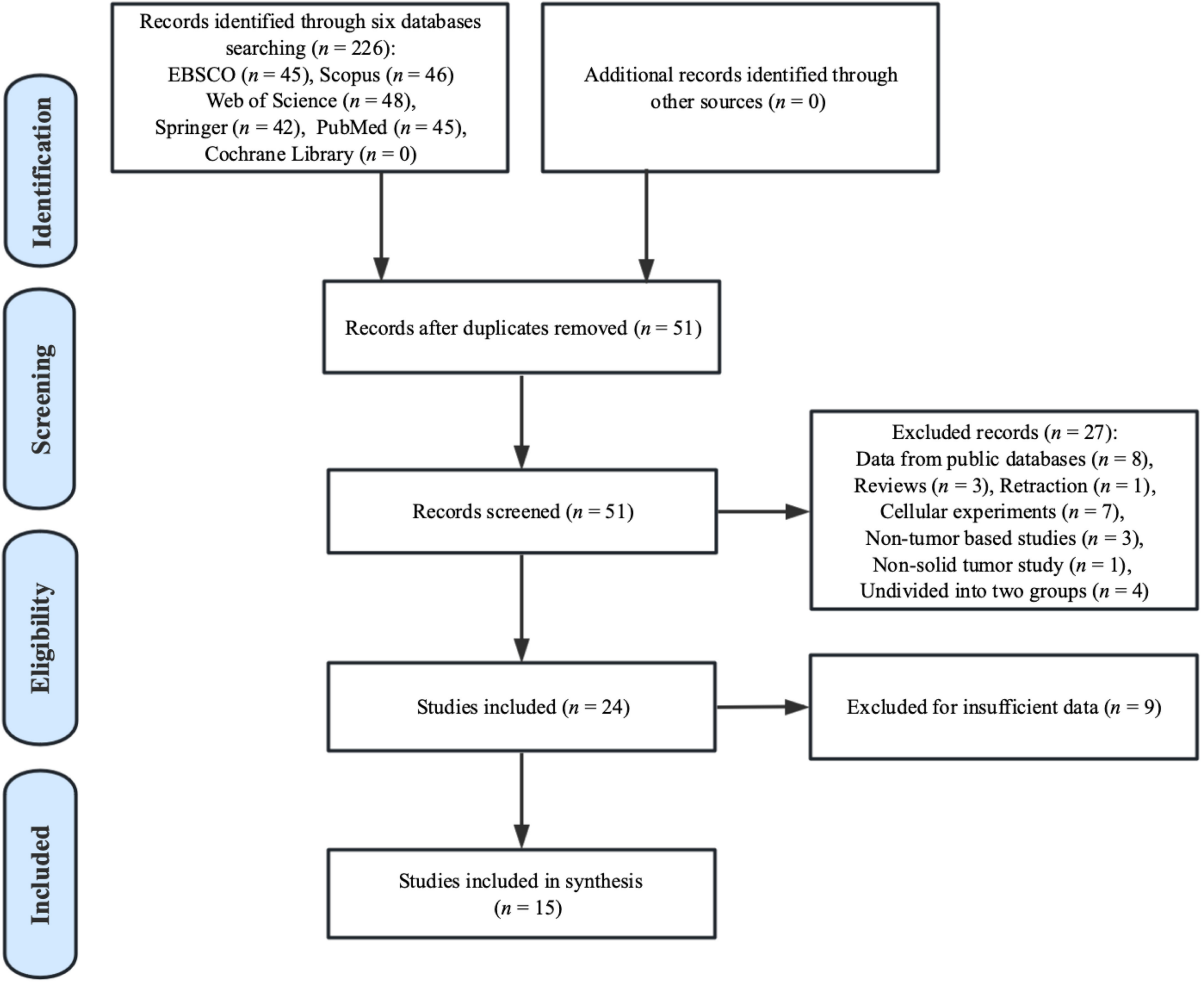
Paper selection flowchart.

**Figure 1** depicts the literature selection process. After the preliminary screening search of six databases, 226 records concerning the association of MAFG-AS1 and cancer prognosis were retrieved. Subsequently, after removing 175 duplicate publications, the remaining 51 studies proceeded to further estimation. We then removed an additional 27 studies as they met the exclusion criteria. Thereafter, 9 records were excluded due to lack of sufficient data after the full-text screening. Finally, 15 studies published between 2018 and 2022 were recruited for the meta-analysis.

### Study characteristics

The characteristics of the 15 included publications are exhibited in **Table 1**. All studies were performed in China with sample sizes varying from 40 to 172 and published from 2018 to 2022. A total of 1187 cases were divided into low and high MAFG-AS1 expression groups. Additionally, qRT-PCR was utilized to determine MAFG-AS1 expression in tissues. Moreover, 12 studies reported clinicopathologic parameters and ten studies offered OS, five of which provided OS data directly. Besides, there were nine kinds of malignant tumors in this meta-analysis, including colorectal cancer, bladder carcinoma, breast cancer, gastric cancer, ESCC, gastric adenocarcinoma, ovarian cancer, hepatocellular carcinoma, and lung adenocarcinoma.

**Table 1:** Main characteristics of included studies.

Author	Year	Cancer types	Sample sizes (*n*)	High expression (*n*)	Low expression (*n*)	Detection method	Outcomes	HR (95% CI) for OS	Follow up (*m*)	Data extraction methods
Cui (22)	2018	CRC	52	27	25	qRT-PCR	CP, OS	2.048 (0.889, 4.711)	NA	directly
Xiao (23)	2020	BUC	102	53	49	qRT-PCR	CP, OS	4.603 (1.708, 12.407)	100	directly
Feng (16)	2020	BRC	50	26	24	qRT-PCR	CP	NA	NA	NA
Li (A) (24)	2020	GC	120	60	60	qRT-PCR	CP, OS	1.756 (1.018, 3.028)	96	directly
Li (B) (14)	2020	BC	43	27	16	qRT-PCR	CP	NA	NA	NA
Qian (25)	2020	ESCC	40	20	20	qRT-PCR	CP, OS	2.61 (0.65, 10.41)	60	indirectly
Sun (15)	2020	BC	52	27	25	qRT-PCR	CP	NA	NA	NA
Cui (26)	2020	CRC	172	86	86	qRT-PCR	CP, OS	1.79 (1.09, 2.95)	60	indirectly
Fu (27)	2021	GAD	60	NA	NA	qRT-PCR	OS	1.67 (0.63, 4.44)	60	indirectly
Tang (28)	2021	BC	66	33	33	qRT-PCR	CP, OS	2.05 (0.86, 4.87)	60	indirectly
Xiang (29)	2021	BUC	102	53	49	qRT-PCR	CP, OS	4.882 (1.791, 13.302)	100	directly
Di (11)	2022	BRC	54	27	27	qRT-PCR	CP	NA	NA	NA
Bai (30)	2022	OC	75	37	38	qRT-PCR	CP, OS	2.18 (0.68, 6.95)	40	indirectly
Tian (31)	2022	HC	152	NA	NA	qRT-PCR	OS	1.697 (1.025, 2.809)	60	directly
Wu (32)	2022	LUAD	47	40	7	qRT-PCR	CP	NA	NA	NA

CRC, colorectal cancer; BUC, bladder urothelial cancer; BRC, breast cancer; GC, gastric cancer; BC, bladder cancer; ESCC, esophageal squamous cell cancer; GAD, gastric adenocarcinoma; OC, ovarian cancer; HC, hepatocellular cancer; LUAD, lung adenocarcinoma; qRT-PCR, quantitative real-time reverse transcription polymerase chain reaction; CP, clinicopathologic parameters; OS, overall survival; HR hazard ratio; CI, confidence interval; NA, not available.

### Association between MAFG-AS1 expression and clinical covariates

**Figure 2:**
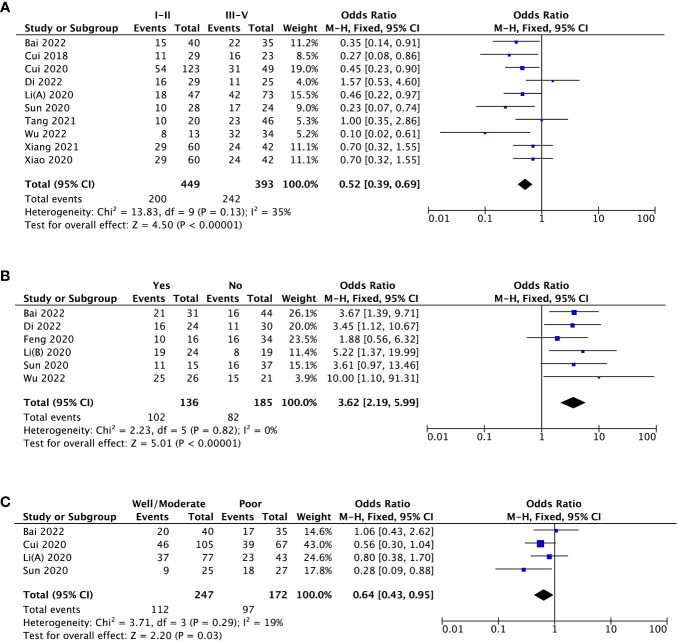
The forest plots assessing the association between MAFG-AS1 expression and clinicopathological parameters [**(A)**, tumor stage; **(B)**, lymph node metastasis; **(C)**, tumor differentiation].

Ten studies estimated the potential association of MAFG-AS1 expression with tumor stage. Due to low heterogeneity among studies (I^2 ^= 35%), the fixed-effects model was applied. As shown in **Figure 2A**, high MAFG-AS1 expression was noticeably associated with advanced tumor stage (P < 0.00001). Regarding the clinicopathologic parameter of LNM, elevated MAFG-AS1 expression substantially predicted LNM (P < 0.00001). Also, the fixed-effects model was used as no heterogeneity was detected (I^2 ^= 0%) (**Figure 2B**). In addition, four studies enrolling 419 cancer patients indicated that high MAFG-AS1 expression was related to worse tumor differentiation (P = 0.03) (**Figure 2C**). Similarly, the fixed-effects model was utilized for low heterogeneity (I^2 ^= 19%). Furthermore, the pooled results were robust after examining with sensitivity analysis.

**Figure 3:**
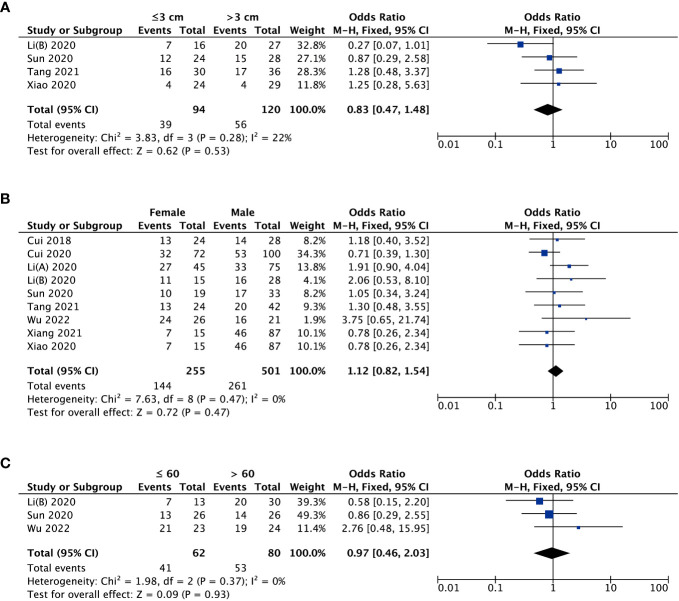
The forest plots assessing the association between MAFG-AS1 expression and clinicopathological parameters [**(A)**, tumor size; **(B)**, gender; **(C)**, age].

**Figure 4:**
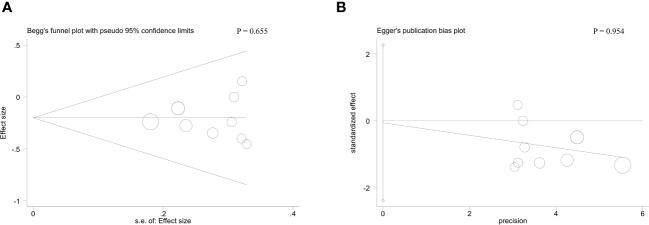
The Begg’s test **(A)** and Egger’s test **(B)** for the association between MAFG-AS1 expression with tumor stage.

With respect to tumor size, data from four studies suggested that there was no striking association between MAFG-AS1 expression and tumor size (P = 0.53) (**Figure 3A**). Additionally, three trial studies in which all patients were female were removed in analyzing the covariate of gender. The result revealed that MAFG-AS1 expression was not correlated with patient gender (P = 0.47) (**Figure 3B**). Also, a total of three studies explored the relationship between MAFG-AS1 expression and patient age (≤ 60 or > 60). The result implied that there was no significant correlation between MAFG-AS1 expression and patient age (P = 0.93) (**Figure 3C**). The fixed-effects model was adopted for all pooled outcomes mentioned above. Simultaneously, the results were not affected by individual studies after sensitivity analysis. Furthermore, Begg’s and Egger’s tests also indicated that there was no publication bias across studies (P > 0.05) (**Figure 4**).

### Association between MAFG-AS1 expression and overall survival

**Figure 5:**
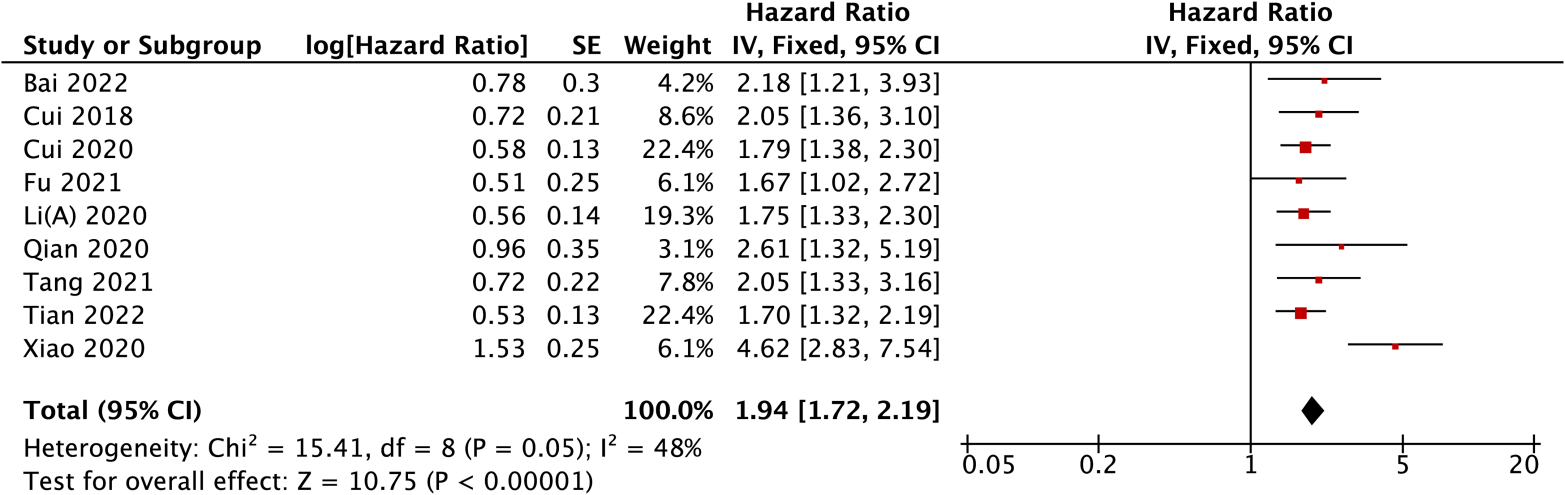
The forest plot for the association between MAFG-AS1 expression with OS.

A total of ten studies were enrolled to analyze MAFG-AS1 expression with OS. Because there was considerable heterogeneity among studies (I^2 ^= 67%); thus, we removed one study (29) using sensitivity analysis, and the heterogeneity was reduced from 67% to 48%. Similarly, the fixed-effects model was conducted. Our results revealed that high MAFG-AS1 expression was significantly related to shorter OS (P < 0.00001) (**Figure 5**). Additionally, the subgroup analysis was performed based on cancer type, sample size, follow-up time, and extracted method. As illustrated in **Table 2**, high MAFG-AS1 expression predicted poor OS in patients with malignancy compared to low MAFG-AS1 expression, regardless of cancer type, sample size, follow-up time, and extracted method (P < 0.05).

**Table 2:** Subgroup analysis of the relationship between MAFG-AS1 expression and OS.

Variables	Total cases (*n*)	HR 95% CI	P	I^2^ (%)	Model
Cancer type					
Gastrointestinal cancer	596	1.80 [1.57, 2.05]	< 0.00001	0	Random
Urologic cancer	270	3.55 [2.00, 6.31]	< 0.0001	77	Random
Sample size (*n*)					
≥ 100	648	2.46 [1.69, 3.58]	< 0.00001	85	Random
<100	293	2.04 [1.63, 2.54]	< 0.00001	0	Random
Follow-up time (*m*)					
> 60	324	3.33 [1.57, 7.04]	0.002	90	Random
≤ 60	565	1.83 [1.58, 2.12]	< 0.00001	0	Random
Extracted method					
Indirectly	413	1.90 [1.58, 2.28]	< 0.00001	0	Random
Directly	528	2.57 [1.71, 3.85]	< 0.00001	84	Random

### Validation of the results based on the GEPIA database

**Figure 6:**
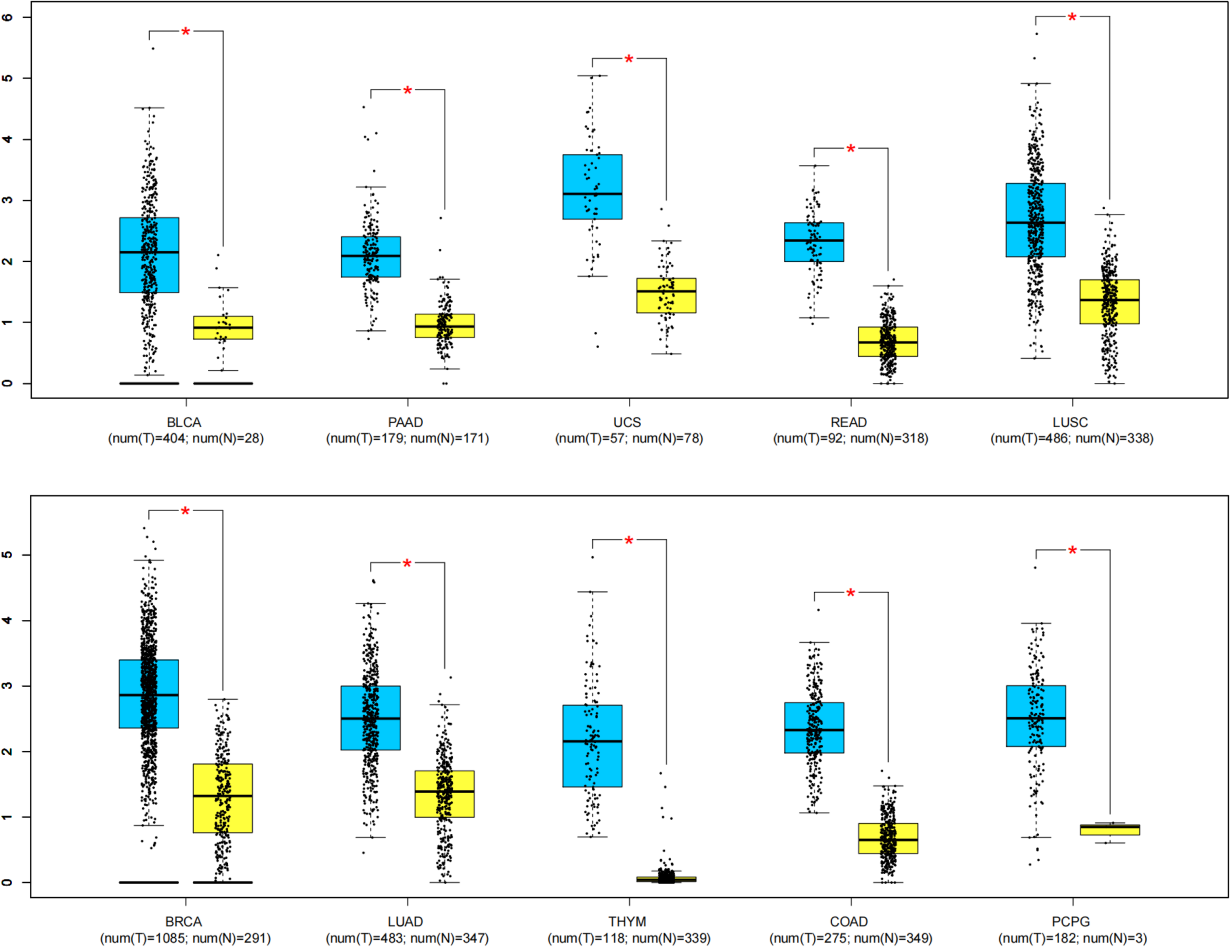
MAFG-AS1 expression in ten kinds of tumor tissues (blue) vs. normal tissues (yellow). “*” P < 0.01. T, tumor tissues; N, normal tissues.

To further strengthen our conclusion, we analyzed the MAFG-AS1 expression in diverse malignancies with the help of GEPIA online gene analysis tool. **Figure 6** indicates that the expression of MAFG-AS1 was dramatically elevated in ten kinds of cancers (P < 0.01), such as bladder urothelial carcinoma (BLCA), pancreatic adenocarcinoma (PAAD), uterine carcinosarcoma (UCS), rectum adenocarcinoma (READ), lung squamous cell carcinoma (LUSC), breast invasive carcinoma (BRCA), lung adenocarcinoma (LUAD), thymoma (THYM), colon adenocarcinoma (COAD), and pheochromocytoma and paraganglioma (PCPG).

## Discussion

With the rapid development of biomedicine, the role of lncRNAs in various physiological and pathological processes of cancer has been gradually clarified, which can even be explored as promising prognostic biomarkers and therapeutic targets for malignancies (33, 34). As a novel lncRNA, MAFG-AS1 is recognized as a vital oncogene since it has intimate terms with unfavorable clinicopathologic parameters and poor prognosis in cancer patients (12). Mechanistically, the underlying cell biological functions behind its role in carcinomas are extremely complex.

First, in urologic tumors, elevated MAFG-AS1 may promote invasion, metastasis, proliferation, and EMT of BLCA via regulation of the HUR/PTBP1 axis, and ultimately contribute to poor OS (23). Similarly, MAFG-AS1 was also shown to activate the PCBP2/FPN1 axis, which then inhibited ferroptosis in BUC cells and subsequently increased cisplatin resistance (29). Besides, Li et al. identified that MAFG-AS1 overexpression repressed miR-143-3p, which further facilitated the proliferation, metastasis, and invasion of bladder cancer cells (14). Also, the same results were confirmed by Sun’s study (15). Meanwhile, cellular experiments proved that MAFG-AS1 could promote the development of bladder cancer by regulating the miR-125b-5p/SphK1 axis (28). Clinically, a lot of patients with bladder cancer struggle to achieve satisfying treatment outcomes due to immunotherapy resistance (35–37). However, there was scarce study investigating the correlation between MAFG-AS1 and immunotherapy resistance. Therefore, it will be a novel direction to explore their relationship in the future. Second, as for gastrointestinal cancers, MAFG-AS1 regulated miR-505 and PLK1 to increase the proliferation rate of gastric cancer cells and decrease the OS of patients with gastric cancer (27). In colon cancer cells, MAFG-AS1 interacted with miR-147b and NDUFA4 and then contributed to cell glycolysis, which was closely associated with cell apoptosis, cycle progression, invasion, and cycle progression (22). In another study, downregulation of MAFG-AS1 could inhibit the invasion, proliferation, migration, and tumorigenesis of colorectal cancer cells via downregulating HOXB8 and upregulating miR-149-3p (38). Overexpression of MAFG-AS1 was established in hepatocellular carcinoma as well. Mechanistic outcomes discovered that MAFG-AS1 accelerated hepatocellular carcinoma cells EMT, migration, and proliferation via miR-3196/STRN4 (39). Third, in breast cancer, hyperactivation of MAFG-AS1 improved the proliferation and migration of tumor cells via modifying the miR-150-5p/MYB axis (40). For hormone receptor-positive breast cancer, MAFG-AS1 could inhibit tumor cell apoptosis and accelerate proliferation via miR-339-5p/CDK2 axis (16), which was consistent with the findings of Li’s study (41). Notably, Gao et al. observed that MAFG-AS1 contributed to the progression and autophagy of breast cancer by regulating miR-3612 and FKBP4 (42). For other types of tumors, Wu et al. recently demonstrated that augmented expression of MAFG-AS1 could promote lung adenocarcinoma cells EMT, proliferation, invasion, and migration by modulating the miR-3196/SOX12 pathway (32). Additionally, Zhao et al. verified that MAFG-AS1 was upregulated in glioblastoma, and that overexpression of MAFG-AS1 inhibited cell apoptosis and facilitated cell migration by affecting miR-34a (43). Taken together, mounting evidence suggested that MAFG-AS1 served an imperative role in tumor development and progression.

This meta-analysis involving 1187 cases and nine kinds of malignancies demonstrated that high MAFG-AS1 expression was obviously correlated with advanced tumor stage (OR = 0.52, 95%CI [0.39, 0.69], P < 0.00001), earlier LNM (OR = 3.62, 95%CI [2.19, 5.99], P < 0.00001), worse tumor differentiation (OR = 0.64, 95%CI [0.43, 0.95], P *=* 0.03), and poor OS (HR = 1.94, 95%CI [1.72, 2.19], P < 0.00001). Nevertheless, there was no remarkable association between MAFG-AS1 expression and tumor size, gender, along with age. Especially, GEPIA database was further adopted to strengthen our results as broadly as possible, and elevated MAFG-AS1 expression was also observed in ten types of cancers, in which six kinds of cancers such as PAAD, UCS, READ, LUSC, THYM, and PCPG were not reported in current published study exploring the significance of MAFG-AS1 with cancer prognosis. Meanwhile, the sensitivity analysis and Begg’s and Egger’s tests supported that our results were robust and reliable. Collectively, our study indicated that MAFG-AS1 can be considered as a novel biomarker for predicting cancer prognosis. Hence, we believe that this meta-analysis will inspire more researchers to investigate the correlation between MAFG-AS1 and malignancy prognosis.

This study has several limitations. First, some studies only provided Kaplan-Meier curves on OS, and thus we indirectly extracted the HR and corresponding 95%CI data utilizing Engauge Digitizer 4.1 software introduced by Tierney et al. (18), which might be inevitably influenced by subjective factors. Second, although we didn’t consider ethnical and geographical restrictions in our records screening procedure, all of the included studies were from China, which, to some extent, might confine the representativeness of the pooled results to other regions outside China. Third, all included studies were from single clinical research; thus, some cutoff values, such as age and tumor size, were inconsistent. Therefore, the most adopted cutoff values were selected for this meta-analysis, which limited our ability to estimate the association between MAFG-AS1 and cancer prognosis with insufficient statistical power. Fourth, though disease-free survival (DFS) is one of the imperative concerns for cancer patients, only one included study explored the correlation between MAFG-AS1 and DFS; therefore, we failed to assess their relationship, which might be an inherent deficiency of this study. Given these limitations above, more high-quality multicenter studies are required to further clarify the significance of MAFG-AS1 in future cancer prognosis.

## Conclusion

In conclusion, this meta-analysis confirmed that MAFG-AS1 is markedly elevated in various malignancies, and that high MAFG-AS1 expression is significantly correlated with advanced tumor stage, LNM, worse tumor differentiation, and poor OS when compared to low MAFG-AS1 expression. Therefore, MAFG-AS1 may be a potential biomarker and can be adopted to accelerate progression against cancer.

## Data availability statement

The original contributions presented in the study are included in the article. Further inquiries can be directed to the corresponding author.

## Author contributions

LG: Conceptualization, Data curation, Formal analysis, Investigation, Methodology, Software, Writing – original draft. LH: Conceptualization, Formal analysis, Investigation, Validation, Writing – original draft. LX: Funding acquisition, Methodology, Resources, Validation, Writing – original draft. LJ: Methodology, Resources, Visualization, Writing – review & editing. XL: Conceptualization, Funding acquisition, Supervision, Validation, Writing – review & editing.
